# Xylidin, Isomere (2,3-Xylidin, 2,5-Xylidin, 3,4-Xylidin, 3,5-Xylidin): MAK-Begründung, Nachtrag

**DOI:** 10.34865/mb8759ismd11_2ad

**Published:** 2026-06-30

**Authors:** Andrea Hartwig, MAK Commission

**Affiliations:** 1 Institut für Angewandte Biowissenschaften, Abteilung Lebensmittelchemie und Toxikologie, Karlsruher Institut für Technologie (KIT) Adenauerring 20a, Geb. 50.41 76131 Karlsruhe Deutschland; 2 Ständige Senatskommission zur Prüfung gesundheitsschädlicher Arbeitsstoffe, Deutsche Forschungsgemeinschaft, Kennedyallee 40, 53175 Bonn, Deutschland. Weitere Informationen: Ständige Senatskommission zur Prüfung gesundheitsschädlicher Arbeitsstoffe | DFG

**Keywords:** Xylidin (Isomere), 2,3-Xylidin, 2,5-Xylidin, 3,4-Xylidin, 3,5-Xylidin, Entwicklungstoxizität, Schwangerschaftsgruppe, Methämoglobin, Hypoxie

## Abstract

The German Senate Commission for the Investigation of Health Hazards of Chemical Compounds in the Work Area (MAK Commission) re-evaluated 2,3-xylidine [87-59-2], 2,5-xylidine [95-78-3], 3,4-xylidine [95-64-7] and 3,5-xylidine [108-69-0] to assess whether the isomers can be assigned to the new Pregnancy Group B (suspected). This group has been recently introduced for substances suspected of causing prenatal tox­icity. Data for prenatal developmental toxicity are not available for any of the xylidine isomers. All four xylidine isomers are indirect methaemoglobin (MetHb) formers. Compared to rodents, humans are far more sensitive to MetHb formation. As with CO-Hb, increased levels of MetHb can lead to impaired oxygen supply to the foetus. Due to their genotoxicity, no MAK value can be derived for the xylidine isomers. Therefore, the assignment to a risk-based pregnancy group is not applicable. As the NOAEC for developmental toxicity by MetHb in humans is not known and the foetus is much more sensitive to MetHb formation than the adult, there is a possibility of endangering the unborn child due to hypoxia. Thus, the xylidine isomers are suspected of causing prenatal toxicity and are assigned to Pregnancy Group B (suspected).

**Table:** 

**MAK-Wert**	**–**
**Spitzenbegrenzung**	**–**
	
**Hautresorption (1966)**	**H**
**Sensibilisierende Wirkung**	**–**
**Krebserzeugende Wirkung (2019)**	**Kategorie 3**
**Fruchtschädigende Wirkung (2025)**	**Gruppe B (Verdacht)**
**Keimzellmutagene Wirkung (2019)**	**Kategorie 3 B**
	
**BAT-Wert**	**–**
	
CAS-Nr.	2,3-Xylidin: 87-59-2 2,5-Xylidin: 95-78-3 3,4-Xylidin: 95-64-7 3,5-Xylidin: 108-69-0

Hinweis: Die Stoffe können gleichzeitig als Dampf und Aerosol vorliegen.

Zu den Xylidin-Isomeren liegen eine Begründung (Greim 1998), ein Nachtrag zur kanzerogenen Wirkung (Greim 2000) sowie eine Gesamt-Reevaluierung (Hartwig und MAK Commission^2020^) vor.

Im Jahr 2024 wurde die Gruppe B (Verdacht) zur Kennzeichnung von Stoffen mit möglicher fruchtschädigender Wirkung eingeführt. In diesem Rahmen werden Stoffe ohne bisherige Schwangerschaftsgruppe auf eine entsprechende Zuordnung geprüft.

## 1 Allgemeiner Wirkungscharakter

Alle vier Xylidin-Isomere führen zur Methämoglobin (MetHb)-Bildung, jedoch in unterschiedlicher Ausprägung. Weitere Details zum Wirkungscharakter sind im Nachtrag von 2020 dargestellt (Hartwig und MAK Commission^2020^).

Ein MetHb-Anteil am Gesamthämoglobin im Blut von über 1,5 % zeigt beim Menschen eine Exposition gegen MetHb-Bildner an. Gesundheitsschädliche Effekte durch MetHb sind bei gesunden Erwachsenen bis zu einem MetHb-Gehalt von 5 % nicht zu erwarten (Leng und Bolt 2008).

## 2 Wirkungsmechanismus

Die MetHb-Bildung geht auf die Kooxidation der im Stoffwechsel entstehenden *N*-Hydroxyderivate und Oxyhämoglobin zurück (Greim 1998). Damit sind die Xylidin-Isomere indirekte MetHb-Bildner.

Beim Menschen ist das fetale Hämoglobin im Vergleich zum adulten Hämoglobin leichter oxidierbar. Das für die Regeneration des funktionstüchtigen Häms aus MetHb verantwortliche Enzym MetHb-Reduktase (Cytochrom-b5-Reduktase) zeigt in seiner Aktivität deutliche Spezies- und Altersunterschiede.

Die Aktivität der NADH-abhängigen MetHb-Reduktase ist in Erythrozyten von Ratten und Mäusen im Vergleich zu denen des Menschen 5- bzw. 10-mal höher. Aufgrund der niedrigeren MetHb-Reduktase-Aktivität beim Menschen sind höhere MetHb-Spiegel die Folge. Beim erwachsenen Menschen ist die MetHb-Reduktase-Aktivität ca. doppelt so hoch wie bei Neugeborenen, was zu höheren MetHb-Spiegeln im Vergleich zu Erwachsenen führt. Bei Ratten und Mäusen sind dagegen die Aktivitäten bei den neugeborenen Tieren deutlich höher als bei adulten. Der Mensch verfügt also im Vergleich zu Ratten und Mäusen über eine viel langsamere MetHb-Regeneration, das gilt sowohl für Erwachsene, besonders aber für Neugeborene (Hartwig und MAK Commission^2025^).

Wie die CO-Hb-Bildung (Hartwig 2015) kann die MetHb-Bildung zur Beeinträchtigung der Sauerstoffversorgung des Fetus führen. Unklar ist jedoch, ab welchem MetHb-Gehalt der Mutter es zu einem Sauerstoffmangel des fetalen Gewebes kommen kann.

## 3 Toxikokinetik und Metabolismus

### 3.1 Aufnahme, Verteilung, Ausscheidung

Zur inhalativen Aufnahme liegen keine Daten vor. Eine Untersuchung mit inhalativer Exposition von Ratten zeigt jedoch eine systemische Wirkung, sodass eine Aufnahme erfolgt. Die Xylidin-Isomere werden nach oraler Gabe gut aus dem Magen-Darm-Trakt resorbiert. Zur Aufnahme über die Haut sind keine Studien durchgeführt worden (Hartwig und MAK Commission^2020^). Die Xylidin-Isomere werden hauptsächlich in metabolisierter Form mit dem Urin ausgeschieden (Greim 1998).

### 3.2 Metabolismus

Der Metabolismus der Xylidin-Isomere ist gekennzeichnet durch Acetylierung oder Oxidation der Aminogruppe, Oxidation einer Methylgruppe bis zur Carbonsäure und Konjugation mit Glycin, Oxidation an freien Positionen des aromatischen Ringes zum Phenol und Konjugatbildung. In geringem Ausmaß kommt es auch zur Bildung von *N*-Methylierungsprodukten (Greim 1998).

Wie bereits im Abschnitt 2 erwähnt, sind die im Metabolismus entstehenden *N*-Hydroxyderivate und Oxyhämoglobin mittels einer Kooxidation verantwortlich für die MetHb-Bildung (Greim 1998).

## 4 Erfahrungen beim Menschen

Zur Reproduktionstoxizität liegen keine Daten vor.

## 5 Tierexperimentelle Befunde und In-vitro-Untersuchungen

### Subakute, subchronische und chronische Toxizität

Im Nachtrag von 2020 ist eine Studie nach OECD-Prüfrichtlinie 407 aufgeführt. In dieser 28-tägigen Schlundsonden­studie an männlichen und weiblichen CRJ:CD (SD)-Ratten kam es mit allen vier Xylidin-Isomeren zur MetHb-Bildung (3,4-Isomer nicht untersucht), hämatologischen Effekten sowie histologischen Effekten auf Leber (Hämosiderinablagerungen), Milz (Hämosiderinablagerungen), Nieren (hyaline Zylinder) und/oder Knochenmark (verstärkte Hämatopoese). Die zuletzt genannten Effekte sind größtenteils als Sekundäreffekte der Wirkungen auf das Blut anzusehen. In Tabelle 1 werden die NOAEL und LOAEL für ausgewählte Effekte aufgeführt. Damit ist ersichtlich, dass hämatotoxische Effekte und deren Folgeerscheinungen bei allen vier Isomeren im Vordergrund stehen (Hartwig und MAK Commission^2020^; MHLW 2018 a, b, c, d).

**Tab. 1:** NOAEL und LOAEL [mg/kg KG und Tag] für ausgewählte Effekte in den 28-Tage-Studien an Ratten (Hartwig und MAK Commission^2020^; MHLW 2018 a, b, c, d)

**Parameter**	**2,3-Xylidin**	**2,5-Xylidin**	**3,4-Xylidin**	**3,5-Xylidin**
NOAEL/LOAEL MetHb-Bildung	12/60	60/300	nicht untersucht	60/360
NOAEL/LOAEL hämatologische Effekte	12/60	12/60	10/50	10/60
NOAEL/LOAEL Organtoxizität	–/12 Milz, Nieren, Knochenmark	12/60 Vormagen, Nieren	10/50 Leber, Nieren	10/60 Milz, Leber, Knochenmark

### Reproduktionstoxizität

#### Fertilität

Es liegen weder Generationenstudien noch Studien zur Fertilität vor.

In der Begründung von 1998 wird beschrieben, dass es Hinweise auf eine Hemmung der DNA-Synthese in den Hoden von Mäusen nach einmaliger Gabe von 2,5-Xylidin (200 mg/kg KG, oral) sowie 3,4-Xylidin (100 mg/kg KG, i.p.) gibt. Zudem hat die 26-wöchige orale Applikation von 125 mg 2,4-Xylidin/kg KG und Tag bei Ratten zu erhöhten relativen Hodengewichten geführt (Greim 1998).

#### Entwicklungstoxizität

Hierzu liegen keine Daten vor.

## 6 Bewertung

Das grundlegende Vorgehen zur Bewertung eines Arbeitsstoffes ist der MAK- und BAT-Werte-Liste zu entnehmen (DFG 2025).

Alle vier Xylidin-Isomere führen zur MetHb-Bildung. Bei Ratten treten nach oraler Gabe auch weitere hämatotoxische Effekte und Wirkungen an Leber, Nieren, Milz und/oder Knochenmark auf.

**Fruchtschädigende Wirkung. **Zur pränatalen Entwicklungstoxizität liegen zu keinem der Xylidin-Isomere Daten vor.

Alle vier Xylidin-Isomere induzieren die Bildung von MetHb, für die der Mensch im Vergleich zur Ratte deutlich empfindlicher ist (siehe Abschnitt 2).

Aufgrund ihrer genotoxischen Wirkung kann für die Xylidin-Isomere kein MAK-Wert abgeleitet werden und die Zuordnung zu einer risikobasierten Schwangerschaftsgruppe entfällt. Da die NOAEC für den MetHb-Gehalt in Bezug auf die entwicklungstoxische Wirkung beim Menschen nicht bekannt ist und der menschliche Fetus MetHb deutlich langsamer regenerieren kann als der Erwachsene, besteht die Möglichkeit einer Gefährdung des Ungeborenen durch eine Sauerstoffunterversorgung. Somit lässt sich ein Verdacht auf eine entwicklungstoxische Wirkung begründen, und für die Xylidin-Isomere wird eine Zuordnung zur Schwangerschaftsgruppe B (Verdacht) vorgenommen.
